# How Medical Studies in Poland Prepare Future Healthcare Managers for Crises and Disasters: Results of a Pilot Study

**DOI:** 10.3390/healthcare8030202

**Published:** 2020-07-09

**Authors:** Patrycja Misztal-Okońska, Krzysztof Goniewicz, Attila J. Hertelendy, Amir Khorram-Manesh, Ahmed Al-Wathinani, Riyadh A. Alhazmi, Mariusz Goniewicz

**Affiliations:** 1 Department of Emergency Medicine, Medical University of Lublin, 20-059 Lublin, Poland; patrycja.okonska@umlub.pl (P.M.-O.); mariusz.goniewicz@umlub.pl (M.G.); 2 Department of Aviation Security, Military University of Aviation, Dywizjonu 303 nr 35, 08-521 Dęblin, Poland; 3 Department of Information Systems and Business Analytics, College of Business, Florida International University, Miami, FL 33174, USA; ahertele@fiu.edu; 4 Institute of Clinical Sciences, Department of Surgery, Sahlgrenska Academy, Gothenburg University, 413 45 Gothenburg, Sweden; amir.khorram-manesh@surgery.gu.se; 5 Research Advisor, Department of Development and Research, Armed Forces Center for Defense Medicine, Gothenburg, 426 76 Västra Frölunda, Sweden; 6 Department of Emergency Medical Services, Prince Sultan Bin Abdulaziz College for Emergency Medical Services, King Saud University, Riyadh 11451, Saudi Arabia; ahmalotaibi@ksu.edu.sa (A.A.-W.); rialhazmi@ksu.edu.sa (R.A.A.)

**Keywords:** crisis management, public health, disasters, COVID-19, climate change, health facility management

## Abstract

In the event of a crisis, rapid and effective assistance for victims is essential, and in many cases, medical assistance is required. To manage the situation efficiently, it is necessary to have a proactive management system in place that ensures professional assistance to victims and the safety of medical personnel. We evaluated the perceptions of students and graduates in public health studies at the Medical University of Lublin, Poland, concerning their preparation and management skills for crises such as the COVID-19 pandemic. This pilot study was conducted in March 2020; we employed an online survey with an anonymous questionnaire that was addressed to students and graduates with an educational focus in healthcare organization and management. The study involved 55 people, including 14 men and 41 women. Among the respondents, 41.8% currently worked in a healthcare facility and only 21.7% of them had participated in training related to preparation for emergencies and disasters in their current workplace. The respondents rated their workplaces’ preparedness for the COVID-19 pandemic at four points. A significant number of respondents stated that if they had to manage a public health emergency, they would not be able to manage the situation correctly and not be able to predict its development. Managers of healthcare organizations should have the knowledge and skills to manage crises. It would be advisable for them to have been formally educated in public health or healthcare administration. In every healthcare facility, it is essential that training and practice of performing medical procedures in full personal protective equipment (PPE) be provided. Healthcare facilities must implement regular training combined with practical live scenario exercises to prepare for future crises.

## 1. Introduction

Rapid economic development combined with urbanization has led to significant human encroachment on the natural environment; this has exacerbated climate change, increasing the severity and frequency of extreme weather events that often result in morbidity and mortality [1,2]. The COVID-19 pandemic has exposed vulnerabilities in healthcare systems globally. It is imperative to review what healthcare managers are currently being taught to determine gaps in the curriculum to better prepare healthcare leaders for future disasters and pandemics.

When crises occur, rapid and effective assistance to victims is essential. Medical assistance is often necessary, and a significant number of victims require coordinated treatment and transport to healthcare facilities. The work of medical facilities cannot be carried out without an appropriate management system. Therefore, managers of healthcare facilities must have the knowledge and skills needed to manage crises effectively [3].

In Poland, public health is the only field of study that prepares potential healthcare managers. In recent years, this field of study has not attracted much interest as it does not have a standardized accredited curriculum or a defined set of competencies for healthcare administrators. In many high-, middle-, and low-income countries, schools of public health have developed competency-based curriculums focused on healthcare administration [4,5,6]. The Healthcare Institutions Law states that a healthcare facility manager is a person who possesses higher education and has proper knowledge and experience to perform managerial duties [7]. The presented requirements indicate that the function of a manager in a healthcare facility can be performed by a person who does not necessarily have prior medical or managerial education specific to healthcare. Management of healthcare organizations is complex. Unlike most industries, unfamiliarity with healthcare operations may result in the director of a healthcare facility being unable to assess the risk of an epidemic or a disaster objectively. In turn, this may result in inadequate management and, consequently, may contribute to negative consequences for patients and staff of the medical facility.

The aim of this study was to evaluate the perceptions of students and graduates in public health studies at the Medical University of Lublin, Poland, concerning their preparation and management skills for crises such as the COVID-19 pandemic.

The standard of the Polish medical training in 2019 may raise some concerns about the preparation of future medical staff in management of mass casualties resulting from major incidents and disasters, as well as an overwhelming amount of sick patients that can stress a healthcare system due to a pandemic. For example, the subject of disaster medicine is only included in the medical rescue course. The preparation of medical students for crises and infectious diseases at the Medical University of Lublin’s Department of Public Health is presented in Table 1.

Public health studies are not regulated in Poland and therefore do not have a specific standard of education. It is the only field of study in Poland that prepares potential healthcare managers. Graduates from the Medical University of Lublin’s Department of Public Health curriculum are taught epidemiology, infectious diseases, and crisis management. During the students’ first level of study (undergraduate studies), they are taught the following subjects: basics of epidemiology, virology, basics of sanitary-epidemiological monitoring, epidemiology, environmental epidemiology, basics of nosocomial infections, and crisis management. At the second level of studies (postgraduate studies), the subjects are disaster medicine, general epidemiology, nosocomial infections, and other threats.

The current COVID-19 pandemic necessitated revisiting the current preparedness in the Polish medical facilities and among medical professionals for the next event. Thus, we aimed to investigate the readiness level among trained professionals in public health with organizational knowledge in management of healthcare services.

## 2. Materials and Methods

We conducted an extensive analysis of the literature. The findings were analyzed using the Nominal Group Technique (NGT) and the acquired knowledge was sorted using categorization and knowledge mapping to develop a questionnaire. Using instant messaging, the respondents had the opportunity to contact and ask questions if necessary. People working in healthcare facilities used this option to clarify the problems they encounter at work connected with the pandemic. An extensive analysis of literature and then the arrangement of the acquired knowledge through categorization and knowledge mapping led to the development of a research tool in the form of a questionnaire. A qualitative method was used to verify the research tool, and the questionnaire was tested on a sample of 6 students to check whether the respondents understood the questions it contained. This group was then excluded from the pilot study and their answers were not included in the final analysis. In the pilot study, an original questionnaire was available in online versions, which contained 6 closed questions and 2 questions that were open and allowed the respondents to express their opinions freely. Statistical and frequency analyses, and review of basic descriptive statistics were conducted using IBM SPSS Statistics Version 26. The study was not a medical experiment and legally did not require the approval of the Bioethics Committee.

## 3. Results

The study involved 55 students and graduates, including 25.5% (14 subjects) men and 74.5% (41 subjects) women. Among the respondents, 41.8% (23 subjects) currently worked in a healthcare facility and 58.2% (students) did not.

Among people working in healthcare facilities, only 21.7% (5 subjects) in the present workplace had participated in preparation training related for mass-casualty incidents and disasters (e.g., epidemics). In comparison, 78.3% (18 subjects) had not undergone such training.

The respondents evaluated their workplaces’ preparedness for pandemics on a Likert scale from 0 to 10. Table 2 presents the subjective evaluations of the respondents. Most respondents rated the preparedness of their workplaces at three points (26%) and five points (26%), followed by 13% for six points, and 9% for zero points or one point. No one rated the preparedness of their workplace at the maximum of 10 points. The highest rating was seven points. However, some of the respondents worked in hospitals. In contrast, others worked in healthcare facilities such as outpatient clinics or primary care practices. In these workplaces, the risk is lower and advanced life-saving equipment, such as respirators, is not required in the workplace.

As many as 96.4% (53 subjects) of the respondents acknowledged that healthcare managers should receive education and training concerning the management of healthcare facilities during emergencies and disasters, only 3.6% (two subjects) were of the opposite opinion.

Respondents were asked what kind of training should be included in the curriculum to develop competences and skills in crisis and disaster management. Similar responses were grouped thematically. Most often, respondents reported the need for practical classes and simulation of crisis events (26.44%), and some reported that these should be theoretical classes (13.79%). They proposed classes on the organization of the work of personnel and human resources management during crisis events (6.90%). They indicated that there should be more classes on the subject of crisis management (6.90%). Furthermore, 6.90% of respondents reported the need for PPE training (learning to put on and take off suits, goggles, visors, and protective masks).

The respondents reported that they never had the chance to put on or see others put on PPE during their studies, so they did not know how to use it properly and safely at work. They reported significant difficulties in performing patient care and the need to practice procedures in full PPE beforehand, as the range of movement in such situations is limited. Only two of the respondents expressed that they did not feel the need for any additional training. Respondent answers are presented in Table 3.

The majority of the respondents (49 subjects) responded that healthcare management students should educate themselves about climate change and its impact on health.

In the second question, where respondents were free to express their opinion by submitting an answer, they were asked to state their concerns if they did not have sufficient skills or competencies to manage crises as a current/future healthcare facility manager. The respondents (25%) were most often afraid that they would not be able to cope with the situation and that they would not be able to predict the development of the situation and make the right decisions. Nearly 15% of the respondents were worried about the lack of experience in such situations, and 13% were afraid that the competencies they had would not be sufficient to manage a crisis. All of the respondents’ answers are presented in Table 4.

## 4. Discussion

Our findings suggested that among the respondents who worked in healthcare facilities, as many as 78.3% had not received any training related to preparation for crises and disaster. Similar research was conducted in Yemen in 2017, where 531 healthcare workers were surveyed using a questionnaire. The analysis showed that the general state of knowledge of Yemeni healthcare workers was insufficient in relation to crisis and disaster preparedness. A total of 41% of all respondents had not participated in any disaster preparedness courses, and 58.9% of respondents had not participated in any practical exercises on crisis and disaster preparedness. Additionally, managers seemed insufficiently qualified in emergency planning and crisis management, as their level of knowledge was lower than that of the medical personnel [9].

In Poland, the first diagnosed COVID-19 patient (patient zero) was recorded on 4 March, 2020 [10], whereas in China, according to official sources, the first patient was diagnosed on 8 December, 2019 [11]. In Poland, during those three months before the coronavirus was diagnosed and reported, people had started to buy mainly protective masks and thermometers. Large quantities of PPE (e.g., protective masks, gloves, thermometers) were exported to China, as there was a huge demand in this region. Due to this, a few weeks before the coronavirus’s appearance in Poland, the amount of available PPE was insufficient. The biggest problem turned out to be the lack of PPE for medical personnel. Until then, hospitals that rarely had to deal with highly infectious diseases did not have a sufficient quantity of protective clothing, visors, or appropriate protective facemasks at their disposal. Managers of healthcare facilities had not anticipated such a problem, most of them had no supplies, and the situation was a complete surprise to them.

Respondents working in the hospitals reported that visitors of hospitalized persons were taking protective gloves and disinfectant supplies out of the wards. A mandatory total ban on visits was introduced shortly after the first COVID-19 patient’s appearance, which brought an end to this practice. Donors and community members came to the rescue, sewing masks and making homemade visors in a spontaneous gesture of help.

An important problem, which the respondents pointed out, was the training on PPE use. Wax and Christian outlined the correct use of PPE for medical personnel, including guidelines on how to safely remove their protective clothing to not expose themselves to secondary contamination. The researchers stressed the need to define clear protocols for cleaning PPE for subsequent use [12]. According to the United States Occupational Safety and Health Administration (OSHA), it is essential for medical personnel to know the requirements for a given emergency [13]. These guidelines should serve as a basis for national protocol development in the standardized use and training of PPE in medical facilities.

Most respondents, when asked to express their concerns about being able to manage a healthcare facility in a crisis situation independently, indicated a lack of experience in such circumstances, a fear that their competencies would prove insufficient, and an inability to predict the development of the situation and to manage it properly. Management in crises is difficult, stressful, and raises several concerns. Unfortunately, a review of Polish and foreign literature on the management of healthcare facilities in crises has shown that there is a paucity of published research in this area [14,15]. A significant problem reported by the respondents is the need for training in operations management and human resource management. Nowadays, in a healthcare organization’s preparation for crises and disaster, the human factor plays a vital role. A shortage of medical personnel, high workloads, an increasing amount of documentation, and the fulfillment of several necessary formalities make medical practitioners reluctant to participate in training [16]. Complacency also plays a contributing factor during a prolonged period of relative calm when no unexpected events occur. In this case, it can be difficult to expect staff to familiarize themselves with the documentation, standard procedures, and plans of mass-casualty incidents and disasters. In most cases, it consists of a quick, cursory review. In times of prolonged periods of security, people do not feel an imminent threat and, therefore, do not have the strong motivation to prepare for a crisis.

The situation related to the spread of COVID-19 is unprecedented and difficult for all managers [17]. The creation of hospital crisis management plans, evacuation plans, or handling of mass-casualty incidents, and the obligation for staff to become familiar with them, is an important step toward preparedness for these events. However, the lack of such plans can lead to chaos and complete disorientation. The effects of disasters can be mitigated by adopting risk management measures and appropriate planning, education, and training measures [18].

The most frequently mentioned need identified by students and graduates in public health was additional training (26.5%), which was based on practical exercises and simulations of real crises. The foundation for effective healthcare operations management should consist of regularly scheduled exercises and training in the procedures related to crises. Skills rehearsed in practice improve preparedness and confidence, allowing one to verify knowledge and analyze the effectiveness of their actions, all of which cannot be achieved using only theoretical training or assumptions [19,20].

A significant number of respondents, 89.1% (49 persons), felt that healthcare management students should receive education about climate change and its impact on health. Many threats result from global climate change. These threats are caused mainly by rapidly developing industries and excessive human expansion into nature, raising the probability of various types of types of extreme weather events and new diseases, including infectious diseases and cancers, and their occurrence is increasing. The World Health Organization (WHO) identified climate change impacts on health and epidemics as the most urgent health challenges in the next decade [21]. Medical and management personnel should be prepared for the possibility of an event that will exceed the local response capacity and lead to excessive morbidity and mortality [22,23,24].

## 5. Recommendations for the Future

The approach to crisis management issues adopted by the manager of a healthcare facility is an essential and decisive aspect. If a healthcare facility’s management approaches the problem of preparedness for crisis incidents seriously, it can force the employees to read the documentation and participate in practical exercises [25]. This can be achieved by organizing several rounds of practical exercises so that all employees can participate. In addition, management should carry out tests to check employees’ knowledge of plans and procedures for dealing with mass-casualty incidents and disasters, evacuation of buildings, and dealing with infectious diseases. If the head of the healthcare facility treats these issues as important, hospital staff will address them in the same manner [20].

Healthcare organizations are challenging to manage on a day to day basis. A crisis event creates additional operational issues that require specialized knowledge and skillsets to manage effectively and safely. Poland should consider increasing healthcare threats to its citizens as a catalyst to introduce new a curriculum in healthcare administration. At the undergraduate and graduate levels, programs can be created that develop competencies in disaster management and crisis leadership to prepare the workforce to manage future healthcare emergencies resulting from epidemics, natural disasters, or man-made events such as terrorism [26,27,28,29,30].

## 6. Limitations

The main limitation of this study was that only one university was surveyed. However, it was a pilot study that revealed gaps in training and education related to crisis and disaster management in the public health studies curriculum, one that is preparing students to manage and lead healthcare organizations. To date, no similar research has been conducted in Poland. This research forms the basis for planned future studies with more respondents and a more comprehensive approach aimed to determine the preparedness and training of healthcare administrators currently managing healthcare facilities in Poland.

Another limitation is the low number of respondents in total. However, as a pilot mixed methods study, the outcome indicates a need for further studies with a larger population.

## 7. Conclusions

Managers of healthcare facilities should have the knowledge and skills to manage crises. They should have an education in public health with core courses in healthcare management. The public health curriculum should also develop competency-based programs that build knowledge and skillsets for managers to deal with emergencies and disasters effectively. Training in PPE use and exercises in performing medical procedures in full protective clothing should be mandatory in all medical facilities. In Poland, regular tabletop and live scenario exercises must be an integral operational consideration for all healthcare facilities to remain prepared for future crisis

Management of major incidents, disasters, and public health emergencies is complex and requires a multidisciplinary approach. Future managers of healthcare facilities should be taught public health and medical knowledge but also be trained to collaborate, cooperate, and communicate effectively during a crisis. Developing a competency-based healthcare management program together with regular tabletops and scenario-based simulation training should be considered to facilitate and improve the future Polish preparedness for disasters and pandemics.

## Figures and Tables

**Table 1:** The 2019 educational standard for medical specializations.

Field of Study	Courses Implemented in a Given Field of Study Related to Preparation for Crisis Events	
	Disaster Medicine, Crisis Management	Infectious Diseases, Epidemiology
**Medical**	-	Infectious diseases
**Medical dentistry**	No specific subjects listed in the regulation, although the listed learning outcomes include knowledge and skills in epidemiology and infectious diseases	
**Nursing**	-	Hospital-acquired infections (undergraduate) Epidemiology (postgraduate)
**Obstetrics**	-	Hospital-acquired infections (undergraduate)
**Laboratory diagnostics**	-	Hygiene and epidemiology
**Physiotherapy**	-	Demography and epidemiology
**Pharmacy**	-	Pharmacoepidemiology
**Medical rescue**	Disaster Medicine	Infectious diseases

Source: Standards of training for medical professions [8].

**Table 2:** Subjective evaluations of the respondents of the preparedness of healthcare facilities in which they work for the COVID-19 pandemic.

Scale from 0 to 10	Respondents’ Evaluations	Respondent Ratings in Percentage
0	2	9%
1	2	9%
2	0	0%
3	6	26%
4	2	9%
5	6	26%
6	3	13%
7	2	9%
8	0	0%
9	0	0%
10	0	0%
Total	23	100%

**Table 3:** Types of training that the respondents believed should be included in their study program.

Responses	Answers	%
Practical training, event simulation	23	26.44%
Regulatory framework for crisis management	4	4.60%
Use of personal protective equipment (PPE); training on how to put on suits, fit goggles, and how to protect yourself to stay safe	6	6.90%
Organization of the work of personnel and human resource management	6	6.90%
Rules of cooperation with other entities	1	1.15%
First aid in the case of infectious diseases	2	2.30%
Communication in crises situations	2	2.30%
Structure of the crisis management system	4	4.60%
Classes in disaster medicine	2	2.30%
Triage	2	2.30%
Theoretical training, lectures	12	13.79%
Education on climate change and the consequent threats	1	1.15%
Psychological support for the people affected by crisis situations and their families	1	1.15%
Gathering and procuring resources	3	3.45%
Making decisions in difficult situations, coping with stress	3	3.45%
Prevention, minimizing the impact	2	2.30%
Working with the media	1	1.15%
Management algorithms	3	4.60%
Crisis management	6	6.90%
I do not feel the need for any training	2	2.30%
**Total**	87	100%

**Table 4:** Concerns the respondents felt about their ability to manage a crisis independently.

Responses	Answers	%
Concerns about the proper functioning of the facility	4	5.88%
Ability to allocate responsibilities well among employees	4	5.88%
The stress of endangering the life and health of a large group of patients and staff	6	8.82%
Fears that their competencies are not insufficient	9	13.24%
Fear that I will not be able to cope, that I will not anticipate the development of the situation, and the fear of chaos	18	25.00%
Concerns about the lack of experience in such situations	10	14.71%
Concern about the lack of specific procedures	5	7.35%
Fear of responsibility and fear that I will make a mistake	6	8.82%
The fear that I will become infected with an infectious disease	1	1.47%
Enormous stress	3	4.41%
No concerns	2	2.94%
**Total**	68	100%
